# Treatment outcomes of Kugel repair for obturator hernias: a retrospective study

**DOI:** 10.1186/s12893-020-00795-8

**Published:** 2020-06-12

**Authors:** Yoshie Hosoi, Hiroshi Asano, Hiroyuki Fukano, Nozomi Shinozuka

**Affiliations:** Department of General Surgery, Saitama Medical University, 38 Morohongou, Moroyama, Irumagun, Saitama, 350-0495 Japan

**Keywords:** Obturator hernia, Kugel repair, Preperitoneal approach, Surgical site infection

## Abstract

**Background:**

We examined the validity and applicability of the Kugel repair approach for obturator hernias, whereby we placed a Kugel patch through the preperitoneal space after placing a short 5-cm skin incision just medial to the anterior iliac spine and 2 cm cranial to the expected origin of the internal inguinal ring.

**Methods:**

We studied patients who underwent surgical Kugel repair for obturator hernias at the Department of General Surgery, Saitama Medical University between 2007 and 2017. We examined the operating time, length of hospital stay, postoperative complications, and mortality rate.

**Results:**

Fifty-eight patients with obturator hernias presented with symptoms of small bowel obstruction. A Kugel approach was used in 53 patients and a midline approach was used in 5 patients with preoperative peritonitis. Of the 53 patients managed using the Kugel approach, 39 did not require intestinal resection; a mesh was used in all these patients. In the remaining 14 patients, intestinal resection was required and performed using the same approach; subsequently, a mesh was used successfully in 6 of these 14 patients. The overall median operating time was 47 min; the corresponding values for procedures with and without intestinal resection were 39 and 68 min, respectively. In terms of postoperative complications, operative mortality was not noted among patients without intestinal perforation; however, superficial surgical site infection developed in 2 patients. Among the 5 patients with preoperative peritonitis who underwent exploratory laparotomy via a midline incision, intestinal perforation was detected during surgery, and all patients required intestinal resection; none of the patients had received a mesh, and 2 patients died after surgery.

**Conclusions:**

The Kugel repair approach was possible even in patients with obturator hernia requiring intestinal resection. However, for patients with perforations, open surgery should be performed after securing the surgical field through a midline incision.

## Background

Obturator hernias develop most commonly in thin older women; they are frequently difficult to diagnose because of the lack of specific symptoms [1]. Intestinal obstruction due to hernia incarceration is the primary symptom, and laparotomy through a midline incision is often selected as the surgical method. Recently, however, various other surgical methods, including inguinal incision [2] and laparoscopic surgery [3–5], have been proposed. This condition is often complicated by prolonged intestinal obstruction and degradation of the general health condition; thus, Kugel repair may be chosen as first-line treatment, considering that a more localized, less invasive approach would be ideal. In this procedure, a Kugel patch is placed from an incision cranial to the internal inguinal ring via the preperitoneal space, not through the inguinal canal. In case of intestinal necrosis, resection can be performed through the preperitoneal Kugel incision. However, this does not apply to patients who have developed perforating peritonitis; in such cases, surgery is performed by creating a midline incision in the lower abdomen, without the use of a mesh to close the hernia orifice. This study aimed to examine the validity and applicability of the Kugel repair approach for obturator hernias based on the patients’ background characteristics, operating time, length of hospital stay, postoperative complications, and mortality rate.

## Methods

### Patients

We retrospectively reviewed the medical records of patients with obturator hernia who underwent surgery at the Department of General Surgery, Saitama Medical University between April 2007 and March 2017. All patients had been diagnosed with obturator hernia based on preoperative computed tomography (CT) findings. CT was performed in all cases to explore the cause of intestinal obstruction; emergent surgery was performed in patients diagnosed with obturator hernia. If the CT scans revealed ascites or free air, or when abdominal findings led to the diagnosis of peritonitis, a midline incision was made in the lower abdomen. For all other cases, Kugel repair was selected.

### Surgical procedure

Kugel repair is a surgical method for inguinal hernias performed via a preperitoneal approach under direct vision, as reported by Kugel in 1999 [6]. A Kugel patch (Bard, Murray Hill, NJ) is composed of 2 polypropylene sheets and a polyethylene polymer memory recoil ring, which allows the mesh to spring back to the original shape after it is folded for insertion; hence, the mesh can be placed without folds.

**Fig. 1:**
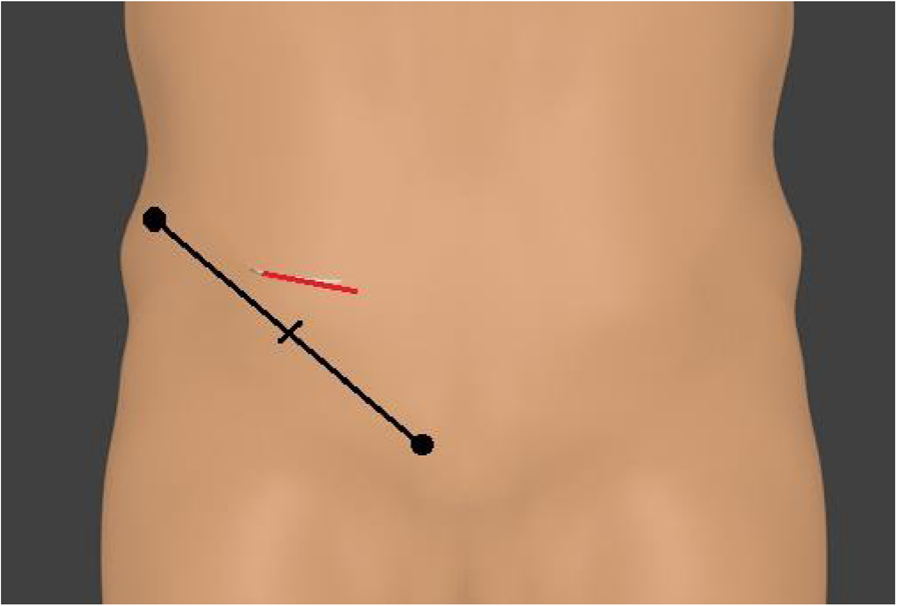
A 5-cm skin incision is made 2 cm cranial to the midpoint between the anterior superior iliac spine and pubic bone

**Fig. 2:**
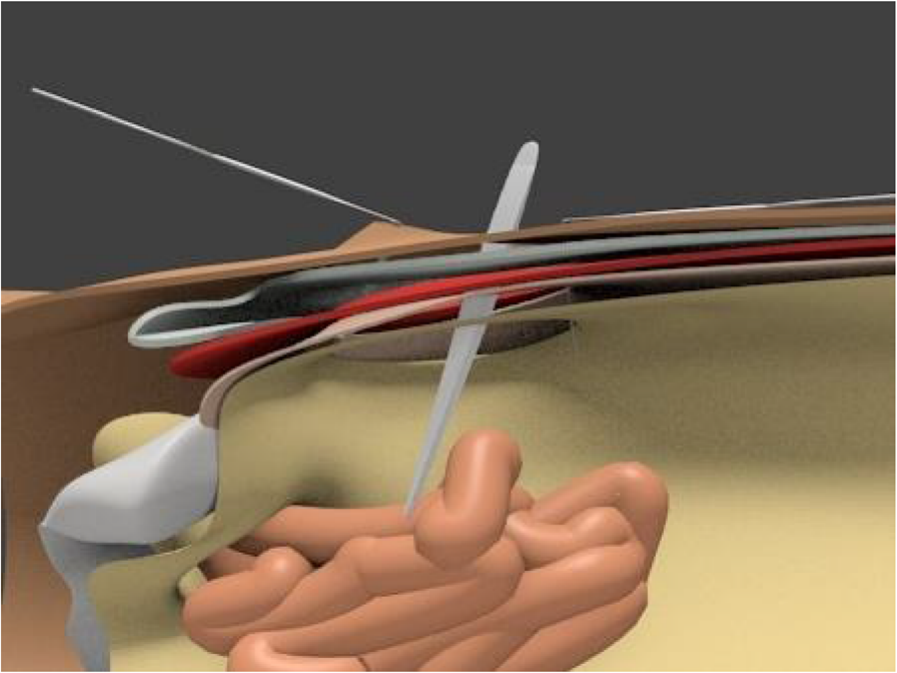
Schema of right obturator hernia from a midline view. The invaginated intestinal tract is reintroduced by manipulation from the abdominal cavity

**Fig. 3:**
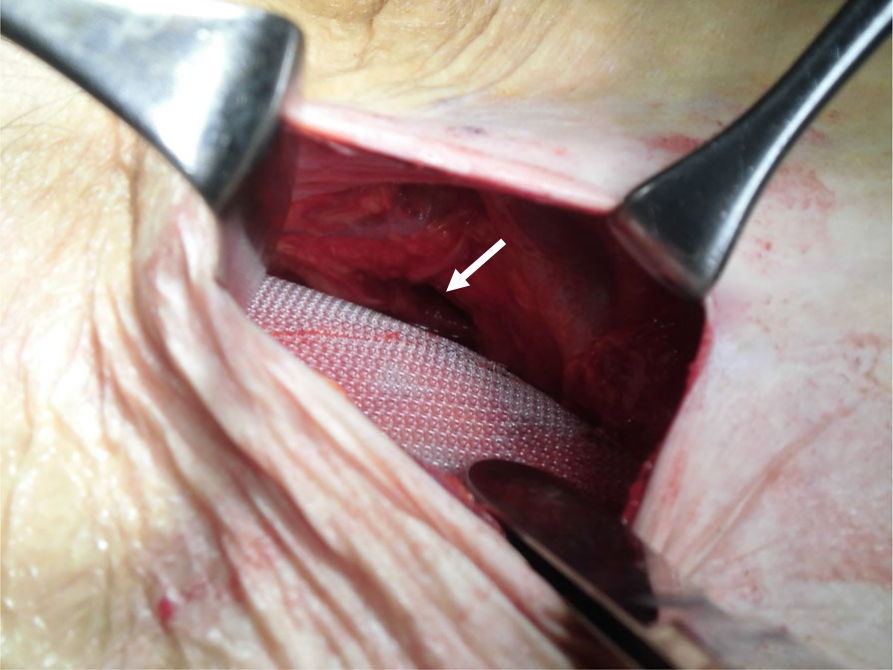
Right obturator foramen, as seen from the preperitoneal space. The mesh is placed to cover the obturator foramen (arrow)

**Fig. 4:**
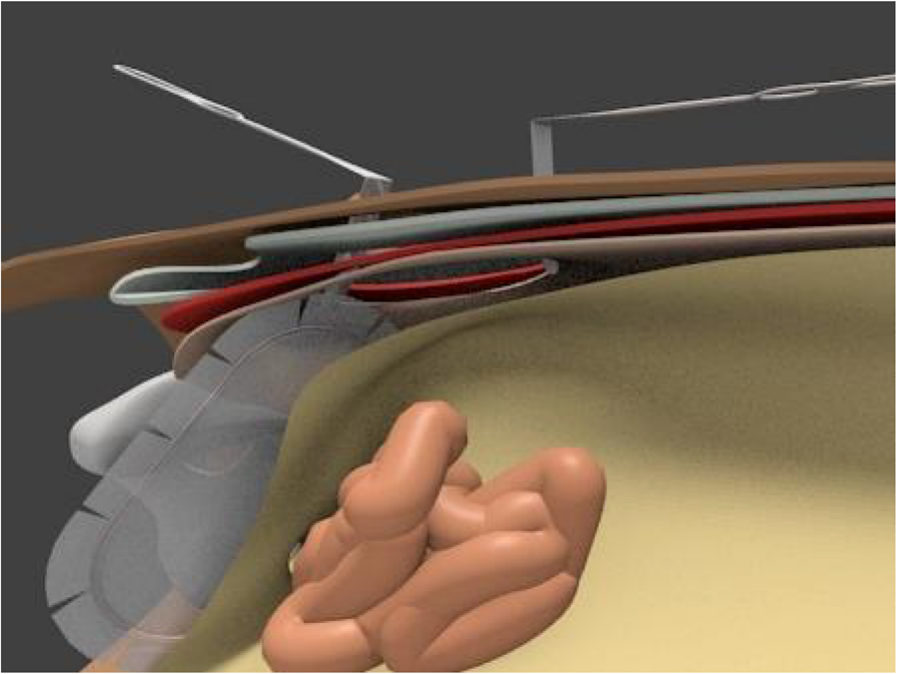
Schema with the mesh placed in the preperitoneal space. The mesh is placed beyond the lower edge of the obturator foramen

Under general anesthesia, a 5-cm transverse incision is made about 2 cm cranially to the location of the internal inguinal ring (Fig. 1), and the preperitoneal space is accessed through the aponeuroses of the external oblique, internal oblique, and transverse abdominis muscles. As most cases correspond to incarcerated hernias, a peritoneal incision is made under direct vision so that the abdominal cavity can be observed to check the condition of the intestines before separating the preperitoneal space (Fig. 2). The incarcerated intestine is reduced from the obturator foramen, and the peritoneum is closed if intestinal resection is not required. Thereafter, the preperitoneal space is gently but bluntly dissected from Cooper’s ligament, the femoral canal, and the obturator canal more posteriorly. The hernia sac extending into the obturator canal is carefully dissected out of the obturator canal if possible, and the area posterior to the obturator canal is also dissected to allow posterior overlap of the mesh to be placed. After excising approximately 3 cm more from the posterior margin of the obturator foramen, an 8 × 12-cm Kugel Patch is inserted (Figs. 3, 4). The mesh is not fixed with sutures to prevent folding. However, if intestinal resection is required because of intestinal necrosis, the edge of the wound is protected using a wound retractor (Alexis® Medical Leaders Co., Ltd., Tokyo, Japan) before the intestine is exteriorized for resection. When there is contamination due to leakage of intestinal fluids during resection, no mesh is used and no treatment is applied to the hernia orifice; only reduction and resection with high ligation are performed in the hernia sac using monofilament absorbent sutures. If it is determined that there is no contamination and the mesh can be placed, the peritoneum is closed before the gloves and equipment are changed to separate the preperitoneal space and place a mesh.

## Results

There were 2293 inguinal hernias during the study period; 58 of these were obturator hernias. The Kugel repair approach was used for 53 patients. Comorbidities included hypertension in 17 cases and heart disease in 12 cases (Table 1).

**Table 1:** Characteristics of patients with obturator hernia

Number of patients	58
Sex (male/female)	1/57
Mean age (years)	82.9 (61–95)
Initial symptoms (multiple possible)	
Vomiting	32
Abdominal pain	22
Lower limb pain	13
Poor appetite	6
Comorbidities	
Hypertension	17
History of abdominal surgery	13
Heart disease	12
Respiratory disease	5

There were 39 patients who did not require intestinal resection; a mesh was used in all these patients. Among the remaining 14 patients requiring intestinal resection, a mesh was used successfully in 6. The overall median operating time was 47 min; the corresponding values for procedures with and without intestinal resection were 39 and 68 min, respectively. Cases of mild intestinal obstruction were discharged on postoperative day 1; however, most patients required additional time for the improvement of intestinal obstruction symptoms, and the median length of hospital stay was 9 days. Bilateral repair was performed in 5 patients who were diagnosed based on preoperative CT and intraoperative findings. Surgical site infection (SSI) was observed in 2 patients; both were cases of surface layer infections. A mesh was used in 1 of these patients because the infection had not reached the mesh. Relapse was observed in 1 patient; however, there were no deaths during hospitalization.

A primary midline incision was used in 4 patients with preoperative generalized peritonitis. In another patient, intestinal perforation was found at the time of opening the peritoneum during Kugel repair, and a second midline incision was required to adequately irrigate the peritoneal cavity. All these 5 patients required intestinal resection because of irreversible ischemia; frank necrosis with perforation was also frequently present. The median operating time and length of stay in such cases were 81 min and 18 days, respectively. SSI was observed as a postoperative complication in 1 patient. Two patients died of perforation peritonitis during hospitalization.

After discharge, patients were followed up at 1 month after surgery; an outpatient visit was scheduled to check the wound. The follow-up was terminated if no issues were found. To date, no mesh-related problems have been detected during the follow-up visits.

## Discussion

Although surgery is, in principle, the treatment of choice for obturator hernias, there is no consensus regarding the surgical method given the variety of access methods. In addition to midline [7] and inguinal [2] incisions, laparoscopic approaches have been increasingly reported in recent years [3, 8]. With regard to the hernia orifice, in addition to the use of a mesh, direct suture closure and coverage of the orifice using internal pelvic organs such as the bladder and uterus have also been reported [9–13]. Patients with obturator hernia are often elderly and have a poor general health condition. Surgery is unavoidable because of intestinal obstruction; furthermore, these patients require general anesthesia by tracheal intubation to prevent the risk of aspiration due to intraoperative vomiting. Therefore, at our institution, we have adopted a preperitoneal approach using a Kugel patch, which only requires a small incision and consequently minimizes invasiveness. Obturator hernias are often incarcerated, with 47–83% cases requiring intestinal resection [14–16]. Thus, safe and adequate intestinal resection is essential for the repair of obturator hernias. With this approach, both intestinal resection and anastomosis could be performed through the same Kugel incision, without the need for an additional skin incision. Furthermore, because the location of the obturator foramen is more dorsal and distal than that of Cooper’s ligament, it is difficult to view the hernia orifice directly from Cooper’s ligament when using an inguinal incision approach [2]. However, with the surgical method used in this study, we approached the hernia orifice through the abdominal oblique muscle group, which is located cranially to the inguinal canal. This provides a better line of sight than does the inguinal incision approach, thereby making it easier to view the hernia orifice directly. Intestinal resection and anastomosis are possible with this operative method. However, because the abdominal cavity must be irrigated in cases of perforation, we believe that open surgery via a midline incision should also be performed. All 5 cases where a midline incision was performed in this study showed perforation, and 2 of these patients died in the hospital; hence, it is important to be aware that the prognosis of obturator hernias with perforation is extremely poor.

Recently, there has been an increasing number of laparoscopic approaches given the advantages, including visibility of the hernia orifice and minimal invasiveness. Obturator hernia can occur on both sides; a previous study reported that 5 out of 8 (62.5%) cases undergoing laparoscopic surgery also had obturator hernia on the opposite side [5]. A significant advantage of laparoscopic surgery is that such an observation is easy to make, and that the same incision can be used to operate on both sides. Nevertheless, despite this method being a minimally invasive approach, the operating time often exceeds 100 min [3, 5]. Moreover, intestinal resection requires extracorporeal manipulation, i.e., an additional small incision is necessary. Bilateral observation is not easy with a unilateral Kugel approach; however, it is beneficial that the treatment can be performed through a small incision in a short time and without the need for the laparoscopic setup. Furthermore, because obturator hernias are more commonly seen in thin women, the obturator foramen on the opposite side can be palpated by inserting a finger into the abdominal cavity through the incision wound. We have also used this method to confirm suspected hernia and operate on the opposite side in cases where incarceration of the intestinal tract was not seen on preoperative CT scans.

A few studies have suggested that the number of complications increases with the use of a mesh to close the orifice in cases of incarcerated hernias without intestinal resection [17–19]. There is no objection to the fact that mesh placement is not indicated in cases of gross contamination [20]. However, the indication for clean-contaminated cases that require intestinal resection is currently under debate. Previous reports suggest that incarcerated inguinal hernias with intestinal resection are not associated with mesh infection; thus, mesh use seems plausible in these cases [21, 22]. We also believe that a mesh can be used in clean-contaminated cases. Nevertheless, in case of intestinal fluid leakage during anastomosis or difficulties with the anastomosis, the use of mesh should be ruled out without hesitation. Furthermore, with this surgical approach, the peritoneum is incised to check the condition of the intestines before separation of the preperitoneal space. If intestinal resection is performed as part of the surgery, the wound is irrigated copiously after closing the peritoneum, and any gloves and equipment used up to that point are changed before proceeding to separation of the preperitoneal space. This is done to divide the mesh placement site and the surgical field for intestinal manipulation, as well as to minimize the risk of mesh infection. To date, no mesh infection has been observed when this technique is used.

## Conclusion

Kugel repair for obturator hernia can be performed by making a small suprainguinal incision and requires a short operating time; therefore, it is recommended for older patients. Furthermore, Kugel repair can still be performed for obturator hernias that require intestinal resection; this surgical technique is also indicated for obturator hernias without perforation. However, cases of perforation require copious abdominal cavity irrigation; therefore, the procedure needs to be performed with a surgical field that allows adequate inspection of the abdominal cavity.

## Data Availability

All datasets generated or analyzed during this study are included in this published article.

## Abbreviations

CT: Computed tomography
SSI: Surgical site infection
